# The Regulation of p53 by Ubiquitination and Implications for Therapeutic Targeting in Colorectal Cancer

**DOI:** 10.3390/genes17030270

**Published:** 2026-02-26

**Authors:** Ioannis A. Voutsadakis

**Affiliations:** 1 Department of Internal Medicine, Division of Hematology, Oncology and Blood & Marrow Transplantation, Holden Comprehensive Cancer Center, University of Iowa, Iowa City, IA 52242, USA; ivoutsadakis@yahoo.com or ivoutsadakis@nosm.ca; 2 Section of Internal Medicine, Division of Clinical Sciences, Northern Ontario School of Medicine, Sudbury, ON P3E 2C6, Canada

**Keywords:** ubiquitination, ubiquitin ligases, ubiquitin conjugating enzymes, proteolysis targeting chimeras, gain-of-function

## Abstract

**Background:** The turnaround of the tumor suppressor p53 protein, the guardian of the genome, is closely regulated to ensure avoidance of its untimely activation, which could lead to the demise of normal cells. Cancer cells often display mutations in the gene *TP53* encoding for p53, which interferes with its normal function. **Methods:** The genomic series of colorectal cancer from the Cancer Genome Atlas (TCGA) was interrogated to discover genomic alterations and determine the mRNA expression of enzymes affecting p53 ubiquitination in colorectal cancers with wild-type and mutant *TP53*. **Results:** Genomic alterations of p53-regulating E3 ubiquitin ligases were uncommon in colorectal cancers, the most frequent being mutations in *RCHY1*. Several p53-regulating E3 ligases were well expressed in subsets of colorectal cancers, two of which, MDM2 and TRIM24, displayed higher mRNA expressions than the normal colorectal epithelia. The former was particularly upregulated in *TP53* wild-type colorectal cancers, and the latter was upregulated in both wild-type and mutant *TP53* cancers. Upregulation of TRIM24 in *TP53* mutant cancers was observed independently of the type of mutations (gain-of-function or other). Among E3 ligases used in proteolysis-targeting chimeras (PROTACs), VHL was upregulated together with its E2-conjugating enzyme UBE2S in colorectal cancers. **Conclusions:** This survey of p53-targeting ubiquitin ligases provides a roadmap for potential therapeutic strategies working by promoting the destruction of the mutant protein or reactivating its normal function in *TP53*-mutated colorectal cancers and promoting p53 function by preventing degradation in *TP53* wild-type cancers.

## 1. Introduction

Proteostasis, the balance between production and degradation of cellular proteins, is an important function of normal cells and is often deregulated in cancer [1,2]. The ubiquitin proteasome system plays a key role in the degradation of aged or dysfunctional proteins, being an integral part of proteostasis. Three enzymatic reactions, catalyzed by three types of enzymes, participate in the ubiquitin tagging of target proteins [3]. The first reaction involves the activation of a ubiquitin molecule by attachment to the E1 enzyme (ubiquitin-activating enzyme). This is followed by transfer to the E2 (ubiquitin-conjugating) enzyme, which then cooperates with the third enzyme, E3 (ubiquitin ligase), which either brings the substrate to the proximity of the E2 and ubiquitin and catalyzes the transfer of ubiquitin to the target proteins or receives ubiquitin from E2 and transfers it to the target proteins. Additional ubiquitin molecules may be linked to the first, creating a ubiquitin chain on the target protein. This post-translational modification may involve different lysines of the ubiquitin protein; a ubiquitin chain of at least four ubiquitin molecules attached to a target protein through the lysine at position 48 (K48) serves as a signal for recognition by the multiprotein complex proteasome for degradation [4].

In colorectal cancer, particularly, some of the most prevalent pathogenic lesions observed, APC mutations are linked with the defective proteasome degradation of transcription coactivator β-catenin [5]. APC is a scaffold protein instrumental in the assembly of the destruction complex that phosphorylates and then ubiquitinates β-catenin in the absence of WNT signals. APC mutations contribute to β-catenin stabilization and subsequent nucleus entry, even in the absence of WNT signaling. In the nucleus, β-catenin cooperates with transcription factor TCF7L2, also known as TCF-4, for the execution of a cancer-promoting transcription program. The destruction complex of β-catenin encompasses the ubiquitin ligase beta-transducin repeat-containing protein (βTrCP), a Really Interesting New Gene (RING) family ligase, which, in addition to β-catenin, targets dozens of other proteins important for carcinogenesis, such as MYC, p53, Snail, Twist, YAP, DEPTOR, Iκ-B, PD-L1, and others [6]. Therefore, depending on the molecular landscape and substrate availability, βTrCP overexpression has been associated with both tumor promotion and suppression [6]. Other genomic alterations of colorectal cancer are also linked to defective proteostasis [7]. For example, MDM2 is a main ubiquitin ligase that degrades tumor suppressor p53 and is induced by kinase AKT, an effector of the receptor tyrosine kinase PI3K/AKT/mTOR signal transduction pathway [8]. p53 is affected by debilitating mutations in about half of colorectal cancers, but increased MDM2 activity may be important for cases with wild-type p53. A further layer of proteostatic regulations is provided by auto-ubiquitination of ligase enzymes or ubiquitination of each other. For example, MDM2 is a substrate of βTrCP. Moreover, receptor tyrosine kinase cascades, which are deregulated by genomic alterations in colorectal cancers, are regulated by proteolysis at multiple levels [9].

Proteolysis-targeting chimeras (PROTACs) are heterobifunctional drug constructs that associate a part targeting a client protein through a connecting unit to an E3 ubiquitin ligase [10]. PROTACs bridge a targeted protein with an E3 ligase, which is part of the PROTAC and ubiquitylates the protein, tagging it for degradation. Thus, target proteins that would otherwise be difficult to inhibit can be eliminated efficiently. Initial efforts to develop PROTAC constructs as anti-cancer drugs had been hampered by problems related to the size of the constructs, tissue and cell penetration, and identification of appropriate targets. Recently, the first PROTACs against nuclear receptors ER and AR have advanced to late clinical development, and the ER PROTAC vepdegestrant has been approved for the treatment of metastatic breast cancer [11]. The effectiveness of these drugs is particularly evident in patients with *ESR1* (the gene encoding for ER) mutations developing under previous hormonal therapy [12]. Several other targets have been used in PROTACs and are currently in clinical development.

Herein, genomic alterations and mRNA expression of the most important ubiquitin ligases involved in colorectal cancer pathogenesis are detailed, based on evaluation of publicly available genomic series. In addition, genomic alterations and expressions of the main ubiquitin ligases that have served as the ligase units of the first PROTAC drugs developed will be interrogated, with an aim of informing optimization of the use of ligases in the development of new PROTACs.

## 2. Methods

RNA and protein expression of p53-associated ligases in a normal colon and rectum were retrieved from the Human Protein Atlas (HPA, www.proteinatlas.org) and the Genotype Tissue Expression (GTEx) dataset [13,14]. HPA is an open database resource that contains data for mRNA and protein expressions in various normal and neoplastic tissues [15]. The Cancer Genome Atlas (TCGA) cohort of colorectal cancers (colorectal adenocarcinoma Pan Cancer Atlas) was evaluated for genomic alterations and mRNA expression of the key E3 ligases involved in p53 regulation [16].

Genetic alterations and expression of important E3 ligases linked to p53 regulation in colorectal cancer were examined using TCGA data. All analyses of alterations in the TCGA cohort were performed using the cBioCancer Genomics Portal (cBioportal, http://www.cbioportal.org) platform, a cancer genomics site maintained by Memorial Sloan Kettering Cancer Center (MSKCC) and other academic institutions [17,18]. The TCGA cohort was analyzed at the level of individual sample clinical characteristics and of individual sample genes and mRNA levels. Whole-genome genotyping was performed with the Affymetrix 6.0 platform. DNA extraction was performed using QiaAmp (Qiagen, Germantown, MD, USA) [16]. TCGA contains data on point mutations, copy number alterations, and structural gene alterations, such as fusions, as well as data on mRNA expressions. mRNA expression quantification was performed in the TCGA series with the RSEM (RNA-Seq by Expectation Maximization) algorithm, which can handle RNA Seq data without a requirement for a reference genome [19]. mRNA levels of expression of genes of interest were extracted using the “mRNA expression z scores relative to normal samples” download functionality in cBioportal. Two scores were used in TCGA studies as indicators of chromosomal instability (CIN). The aneuploidy score (AS) was produced for each sample by adding the number of chromosome arms that had copy number alterations (gains or losses). Copy number alterations were defined as gains or losses in more than 80% of the total length of the chromosome, as measured by the ABSOLUTE algorithm from Affymetrix 6.0 SNP arrays [20]. Chromosomal arms with somatic copy number alterations extending from 20% to 80% of the total chromosome arm length were considered indeterminate and therefore subtracted from the denominator of the AS calculation. Chromosomal arms with somatic copy number alterations covering less than the cut-off of 20% of the arm length were counted as not altered. The second metric of CIN in TCGA, the fraction genome altered (FGA), was defined as the total length of segments with log2 greater than 0.2 divided by the total length of all segments measured in the same sample within source segment files. Extensive quality controls were in place to ensure the quality of specimens included in the TCGA cohort. Samples were required to contain an average of 60% of tumor cells and less than 20% necrosis [16]. Availability of at least 6.9 μg of tumor DNA and 4.9 μg of normal DNA was required for sample inclusion. Stained slides used in the study were reviewed by a board-certified pathologist to confirm the diagnosis of colorectal adenocarcinoma. All *TP53* mutations present in the TCGA cohort were included in the current analyses. Most (98.2%) were considered oncogenic or likely oncogenic, and only 6 of the total 332 mutations in the gene (1.8%) were considered to be of unknown significance. In the functional analysis of *TP53* mutations, samples with more than one mutation were included in the gain-of-function (GOF) group if at least one mutation was GOF.

Gene promoters of interest were identified in the Eukaryotic Promoter Database (EPD, http://epd.expasy.org/epd/ (accessed on 7 January 2026)) using the human genome and restricting the sequences to the −1000 position from the transcription start site to the 100 position [21]. The cut-off *p*-value was set at 0.001. Transcription factor binding motifs were retrieved from the JASPAR Core 2018 vertebrate database [22].

Statistical comparisons were carried out with Fisher’s exact test or *χ*^2^ test for categorical data. Continuous parameters were compared with Student’s *t* test. Kaplan–Meier survival curves were constructed from survival data, and the log-rank test was employed to compare Kaplan–Meier curves. All statistical comparisons were considered significant at *p* < 0.05.

## 3. Results

A panel of eight E3 ubiquitin ligases with evidence of involvement in direct p53 regulation, MDM2, UBE3A (also called E6-AP), HUWE1 (also called ARF-BP1 or Mule), RCHY1 (also known as PIRH2), COP1, TRIM24, RNF34 (also called CARP-1), and RFFL (also called CARP-2), was selected for evaluation. Six of the eight belonged to the RING ligase type, while UBE3A and HUWE1 are HECT (homologous to E6-AP C terminus) type ligases (Table 1). Ubiquitination-targeted lysines are mostly located in the carboxyterminal part of p53, which is a 393 amino acid protein. The eight E3 ligases showed comparable mRNA expression between normal colon and rectum epithelia in HPA and GTEx, with the exception of HUWE1, which displayed higher expression in colon than in rectum epithelia (Table 2). mRNA expression variability between the different ligases was observed, with MDM2, UBE3A, HUWE1, and RFFL showing the highest expressions. At the protein level, among the six p53 ligases with protein expression data in normal colon and rectum glandular epithelia in HPA, high expression was observed by MDM2, UBE3A, and RFFL, while HUWE1, RCHY1, and TRIM24 showed lower expression.

Expression of the p53 ligases was similar in colon and rectum tissues at the mRNA level, with MDM2, UBE3A, and HUWE1 showing the most robust expression (Table 3). At the protein level, MDM2 was assayed with three different antibodies, two of which showed uniformly high expression in all samples tested, while the third showed medium-to-high expression in two-thirds of the samples (Table 3). UBE3A displayed high expression with one of the three antibodies assayed, but moderate-to-low expression in all samples with the two others. HUWE1 was also expressed at moderate or low levels with both antibodies tested. RCHY1 and TRIM24 were tested with one antibody each and were expressed in moderate-to-low levels in about half the samples, while some samples displayed no expression. RFFL was tested with three antibodies, and two showed mostly moderate-to-low expression, while most samples displayed high expression with the third antibody. COP1 and RNF34 were not tested in the HPA.

**Figure 1:**
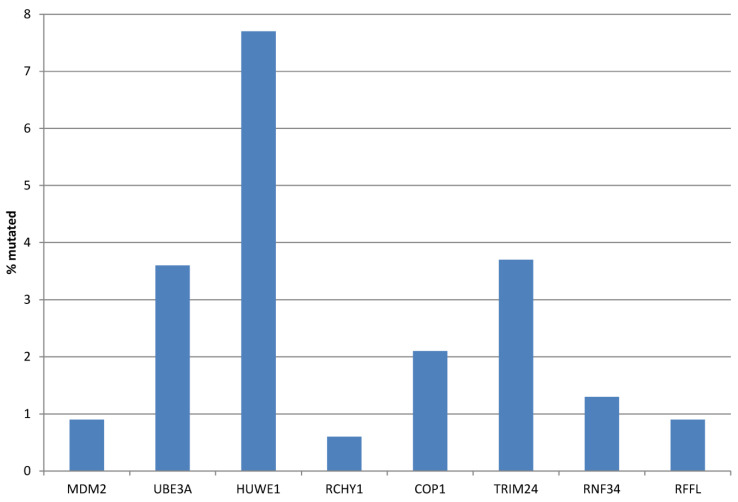
Prevalence of mutations in genes encoding for E3 ubiquitin ligases regulating p53 in colorectal cancers. Data are from TCGA.

The prevalence of genomic alterations in the eight E3 ligases was low in the TCGA colorectal cancer cohort. The most commonly mutated ligase was HUWE1 (7.7% of cohort cases), followed by TRIM24 (3.7%), UBE3A (3.6%), and COP1 (2.1%), and the other four ligase genes were mutated in 0.6% to 1.3% of colorectal cancers (Figure 1). Similarly, copy number alterations were rare, with a prevalence of 1% or lower for all eight ligases.

**Figure 2:**
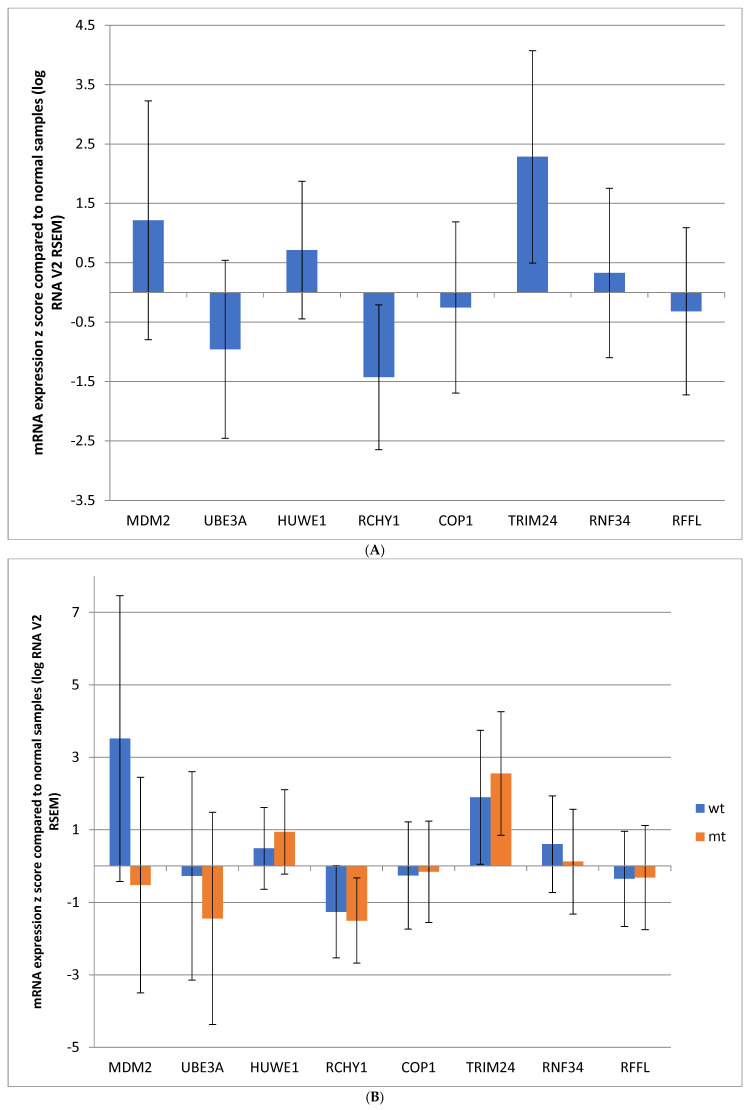
Mean mRNA expression (mRNA z score compared with normal samples) of E3 ubiquitin ligases regulating p53 in colorectal cancers in: (**A**) the entire TCGA cohort; and (**B**) subgroups with wild-type (wt) *TP53* and mutant (mt) *TP53*.

mRNA expression of the eight ligases in the TCGA colorectal cancer cohort was variable compared to adjacent normal tissues. TRIM24 and MDM2 mRNAs were upregulated, and RCHY1 and UBE3A were downregulated; the other four ligases showed no significant differences compared to normal tissues (Figure 2A).

*TP53* mutations are prevalent in colorectal cancers, and among the 534 cases with mutation data in the TCGA colorectal cancer cohort, 314 patients (58.8%) had mutations in *TP53*, the second most prevalent mutated tumor suppressor in these cancers after *APC*. Compared with the group with wild-type *TP53*, *TP53*-mutated cancers showed no significant differences in mean age or gender, but they were of a more advanced stage (51% stages III and IV versus 31.9% in wild-type cancers, *p* = 0.002; Table 4). Moreover, *TP53*-mutated cancers were more frequently located in the rectum (31.7% of mutated cases versus 17.8% of wild-type cancers, *p* = 0.0004; Table 4). CIN and high AS and FGA indices were more prevalent in *TP53*-mutated cancers, while MSI and high tumor mutation counts were more prevalent in cancers with wild-type *TP53* (Table 5). Ligase HUWE1, which displayed the highest prevalence of mutations among the eight p53-related ligases, showed a higher prevalence of mutations in the group with wild-type *TP53* (10.9%) compared to the group with *TP53* mutations (5.4%, Fisher’s exact test, *p* = 0.02).

mRNA expression of the eight p53-related ligases showed notable differences in each case according to *TP53* status (Figure 2B). MDM2 displayed significant upregulation in the *TP53* wild-type group compared to normal samples, while in the *TP53* mutant group, the expression of MDM2 was slightly suppressed, consistent with the importance of p53 function for MDM2 regulation. UBE3A expression was not significantly different in the *TP53* wild-type group compared to normal samples, but was downregulated in *TP53* mutant colorectal cancers. TRIM24 expression was upregulated in both groups independently of *TP53* mutation status and was the most upregulated p53-related ligase in the *TP53* mutant group (Figure 2B).

To explore the regulation of TRIM24 expression, TRIM24 promoter binding sites for important colorectal cancer transcription factors were evaluated in the EPD database, which lists two TRIM24 promoters. Regulation of the two TRIM24 promoters was similar and revealed clustered binding sites for the transcription factor AP-1, a dimer of FOS, and JUN family transcription factors activated downstream of the RAS/RAF/MAPK cascade, as well as clustered sites for the hindgut specification transcription factor CDX2, which is expressed in approximately 90% of colorectal cancers, and hepatocyte nuclear factor 1A (HNF1A), also expressed in colorectal cancers (Table 6). In contrast, the transcription factor TCF7L2 of the WNT pathway, transcription factors TEAD1 and TEAD4 of the Hippo pathway, and the cell cycle-regulating E2F1 factor displayed no clustered binding sites in TRIM24 promoters (Table 6). p53 possessed two binding sites in both promoters, suggesting a weaker influence on their regulation than AP-1, CDX2, and HNF1A.

**Figure 3:**
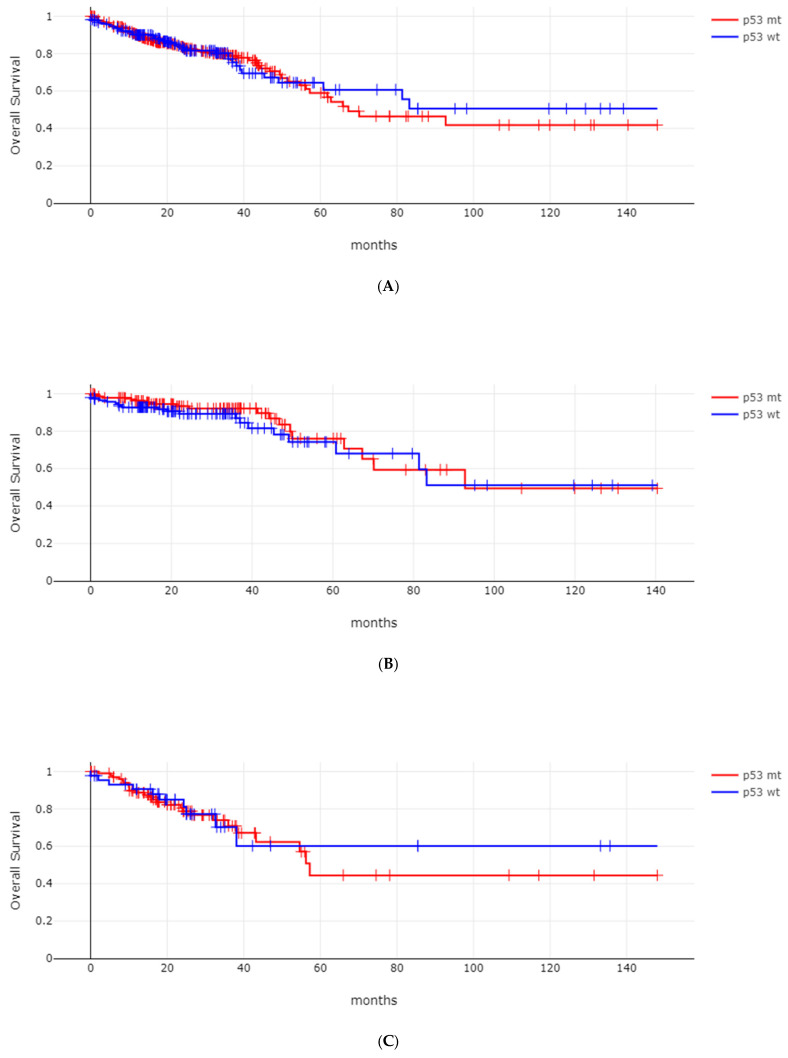

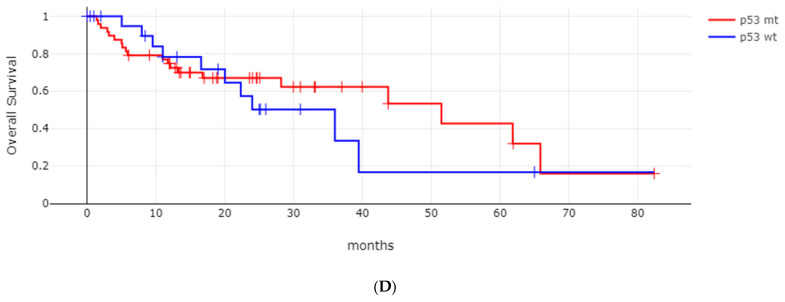
Overall survival (OS) of colorectal cancers according to *TP53* status. (**A**) Entire TCGA colorectal cancer cohort, log-rank test *p* = 0.84. (**B**) Stages I–II: log-rank test *p* = 0.39. (**C**) Stage III: log-rank test *p* = 0.77. (**D**) Stage IV: log-rank test *p*= 0.46. p53 mt: *TP53*-mutated group; p53 wt: *TP53* wild-type group.

Overall survival (OS) of patients with *TP53* mutations did not differ significantly from those with wild-type *TP53* (log-rank *p* = 0.84, Figure 3A). In addition, the OS of the two groups did not differ when categorized according to disease stage (log-rank *p* = 0.39 for stages I and II; *p* = 0.77 for stage III; *p* = 0.46 for stage IV, Figure 3B–D).

**Figure 4:**
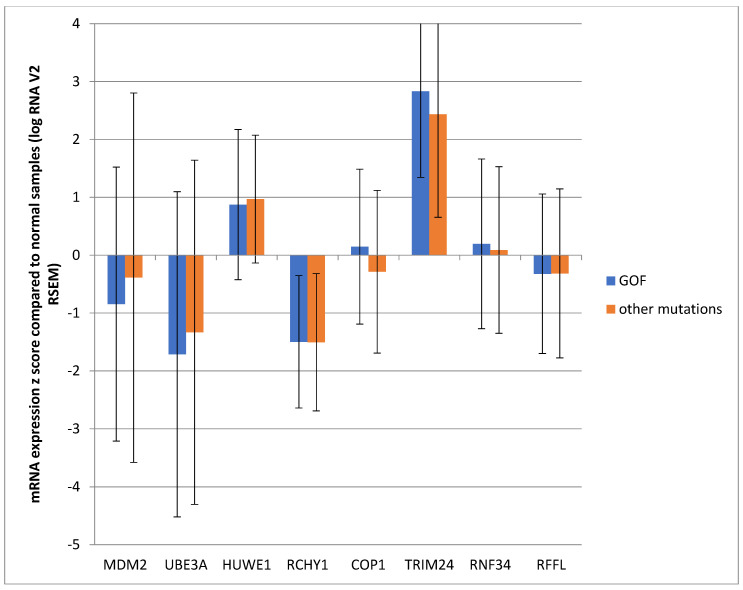
Mean mRNA expression (mRNA z score compared with normal samples) of E3 ubiquitin ligases regulating p53 in colorectal cancers according to the type of *TP53* mutations. GOF: group with gain-of-function mutations; other mutations: group with mutations not included in the GOF group.

**Figure 5:**
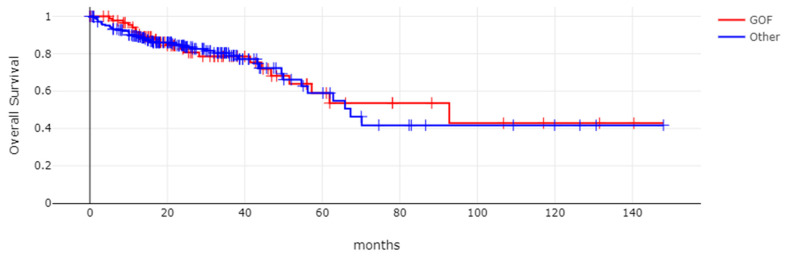
Overall survival (OS) of *TP53*-mutated colorectal cancers according to *TP53* mutation type. Log-rank test *p* = 0.83. GOF: group with gain-of-function mutations; other: group with mutations not included in the GOF group.

To further evaluate the influence of the specific *TP53* mutations on the expression of p53-related E3 ligases, *TP53* mutations were categorized as gain of function (GOF), including R175H, R248W, R248Q, R273H, and R282W, versus other *TP53* mutations. Among the group of 314 colorectal cancers with *TP53* mutations, 94 patients had GOF mutations and 220 patients had other mutations (Table 4). The two different *TP53*-mutated groups did not differ significantly in their age, tumor location, stage, or molecular subgroups (Table 4 and Table 5). The expression of the eight p53-related ligases’ mRNA was similarly regulated in the groups of colorectal cancers with GOF and other mutations, with MDM2, UBE3A, and RCHY1 downregulated and TRIM24 upregulated in both groups compared with normal samples (Figure 4). The OS of patients with GOF *TP53* mutations did not differ from that of patients with other types of *TP53* mutations (log-rank *p* = 0.83, Figure 5).

**Figure 6:**
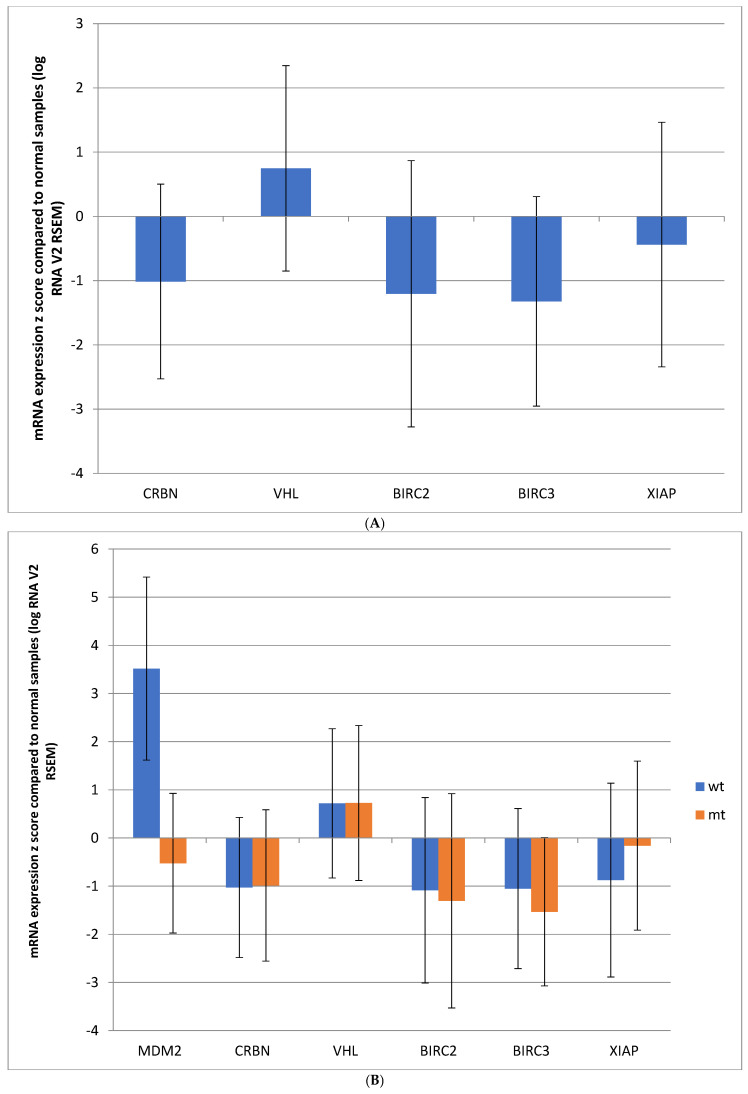

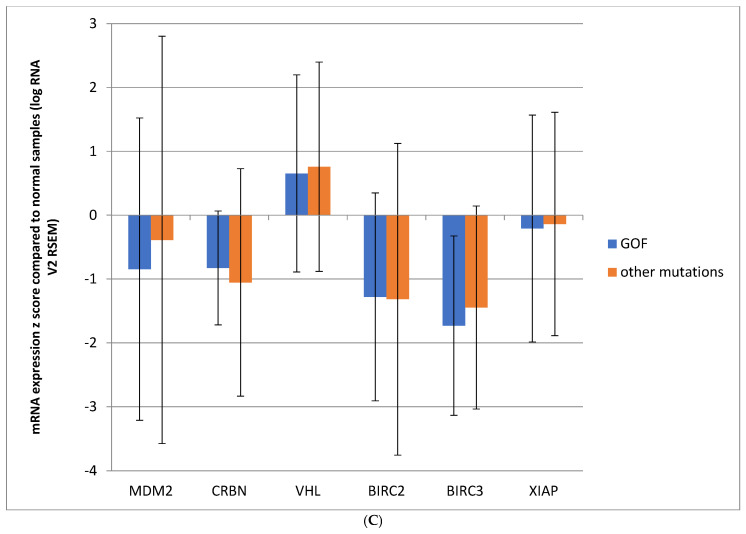
Mean mRNA expression (mRNA z score compared with normal samples) of E3 ubiquitin ligases frequently used in PROTAC constructs in: (**A**) the entire TCGA cohort; (**B**) subgroups with wild-type (wt) *TP53* and mutant (mt) *TP53*; and (**C**) groups with GOF or other *TP53* mutations.

In addition to MDM2, E3 ligases that are used frequently as part of PROTAC drug constructs include CRBN, VHL, BIRC2, BIRC3, and XIAP. None of these ligases were frequently mutated or copy number-altered in colorectal cancers. Mutation rates ranged between 0.7% for VHL and 2.1% for BIRC3 in the TCGA colorectal cancer cohort, and only rare cases had amplifications or deletions. The mRNA expression of these ligases was downregulated compared to normal colorectal tissues, except for VHL, which was slightly upregulated, suggesting that these ligases do not play a pivotal role in colorectal carcinogenesis (Figure 6A). Other than MDM2, mRNA expression of the PROTAC ligases did not differ significantly in *TP53* wild-type and mutant groups, implying that none are regulated by p53 (Figure 6B). Moreover, no significant differences were observed in mRNA expression between the group with GOF *TP53* mutations and the group with other *TP53* mutations (Figure 6C).

**Figure 7:**
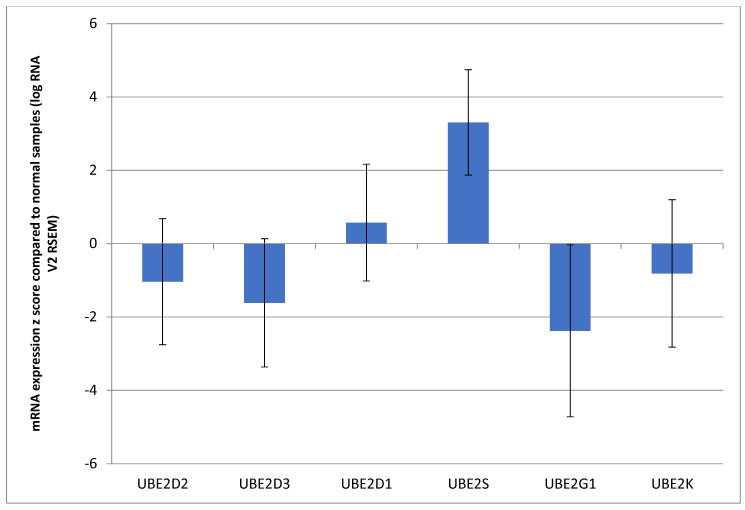
Mean mRNA expression (mRNA z score compared with normal samples) of E2 ubiquitin-conjugating enzymes cooperating with ubiquitin ligases frequently used in PROTAC constructs.

In contrast to the E3 ligases that are part of the PROTAC construct, the E2-conjugating enzymes that work with them to upload the ubiquitin molecules for transfer to the target protein are endogenous to the host cell; therefore, their level of expression may be critical, constituting the bottleneck for the effectiveness of PROTACs that include the respective E3 ligase enzymes. E2-conjugating enzymes working with ligases frequently used in PROTAC constructs include UBE2D2 (also called UBCH5B), UBE2D3 (UBCH5C), UBE2D1 (UBCH5A), E2 enzymes for MDM2, UBE2S (also called E-EPF) working with VHL, UBE2D3, UBE2G1 working with CRBN for the initial ubiquitin upload and the chain extension, respectively, UBE2D3, E2 enzyme for BIRC2, and UBE2K, the conjugating enzyme for XIAP. Among these E2 enzymes, none were frequently mutated or copy number-altered in colorectal cancers. In the TCGA colorectal cancer cases, mutations in these E2 enzymes were observed in one to three cases (0.2–0.6%), except for UBE2K, which had mutations in seven cases (1.3%). Similarly, copy number alterations were rare, observed in none to two cases for five of the six E2-conjugating enzymes, while UBE2G1, encoded from the chromosome arm 17p13.2, displayed deletions in seven cases (1.2%). Mirroring its E3 ligase VHL, E2-conjugating enzyme UBE2S was the only E2 enzyme among the six with significant mRNA upregulation in colorectal cancers compared with normal tissues (Figure 7). The mRNA upregulation of UBE2S was similarly independent of the *TP53* mutation status and, among *TP53*-mutated cases, independent of the type of *TP53* mutations (GOF or other).

Consistent with the lack of p53 dependence, the promoter of UBE2S did not possess p53 motifs. Cluster sites for the WNT/β-catenin pathway transcription factor TCF7L2 and for the transcription factors SMAD2/SMAD3/SMAD4 of the TGFβ pathway were present (Table 6).

## 4. Discussion

Ubiquitination, the attachment of the protein ubiquitin to target proteins, is a versatile post-translational modification that results in a variety of outcomes for targets, and includes trafficking to different cellular compartments and degradation through the lysosome or the proteasome [28]. Ubiquitin attachment is accomplished through a series of enzymatic reactions, which are performed by three types of enzymes: E1, E2, and E3. There are only two E1 enzymes for ubiquitin, UBA1 and UBA6, in human cells, a few dozen E2 enzymes, and several hundred E3 enzymes [29]. UBA1 E1-activating enzyme works exclusively with ubiquitin, while UBA6 also activates the ubiquitin-like protein FAT10. E1 enzymes attach ubiquitin through a thioester bond to the conjugating E2 enzymes, which then transfer ubiquitin to the target proteins in reactions catalyzed by E3 ubiquitin ligases. Depending on the type of ligase, the transfer of ubiquitin from the E2 enzyme to the target is direct or through an intermediate step that transfers ubiquitin onto the ligase before the final transfer to the target protein [30]. Additional ubiquitin molecules may then be added to the first in a similar enzymatic cascade using different lysines of the ubiquitin molecule as points of attachment. Chains through lysine 48 are the canonical signals for proteasome recognition and degradation of the attached protein. Ubiquitin may also form chains through other lysines of the molecule, including at positions 6, 11, 27, 29, 33, and 63, as well as the amino-terminal methionine, some of which also lead to recognition by the proteasome and degradation, but also have alternative outcomes regulating signal transduction, cell cycle, and trafficking [31].

Ubiquitin E3 ligases are classified into three families: RING type (the most abundant), HECT type, and RING-between-RING (RBR) [32]. RING-type ligases function by bringing the ubiquitin-loaded E2 enzymes in contact with the target protein and directly transferring ubiquitin to the target [33]. In contrast, HECT-type ligases form an intermediate ubiquitin bond, receiving ubiquitin from the E2 enzyme and then transferring it to the target protein. RBR-type ligases have a hybrid mode of action that requires activation by E2 loaded with ubiquitin through an interaction with the RING1 domain, and then forms a bond with the ubiquitin molecule through the RING2 domain before transferring it to the target [33]. Both RING- and HECT-type ligases were represented among the p53 regulators examined in the current study.

The data presented here confirm that several E3 ligases involved in p53 regulation, including MDM2, UBE3A, and RFFL, were well expressed in normal colon and rectum epithelia, and others (HUWE1 and TRIM24) had moderate expression. In addition, although some heterogeneity exists, MDM2, UBE3A, and RFFL retained high expression in colorectal cancers, and HUWE1 and TRIM24 showed moderate expression in several colorectal cancers. At the mRNA level, MDM2 showed exclusive upregulation in p53 wild-type cancers, while TRIM24 was upregulated in both p53 wild-type and mutant colorectal cancers. TRIM24 showed the most significant upregulation in p53-mutated colorectal cancers, and this was independent of the type of *TP53* mutations (GOF or other). Among ligases frequently used as PROTAC components, VHL was moderately upregulated, together with its cooperating E2-conjugating enzyme UBE2S, while other frequently used ligases in PROTACs were downregulated. These results have implications for the rational development of PROTACs with increased activity in subsets of colorectal cancers with or without *TP53* mutations.

Allton et al. identified the tripartite family protein TRIM24 as an interacting partner of p53, leading to p53 ubiquitination and degradation [34]. Knock-down of the homolog bonus in drosophila cells led to increased apoptosis, which was rescued by knock-down of p53. Another study suggested that TRIM24 is also induced by p53 and auto-destructs after phosphorylation by the kinase ATM during the DNA damage response, coordinating p53 stability [27]. In addition, TRIM24 is able to recognize a combined histone H3 modification signal consisting of unmethylated lysine 4 and acetylated lysine 23 (H3K4me0/H3K23ac), which is a transcription activation module and is recognized by a tandem array of a PHD domain and bromodomain of TRIM24 [35]. Acetylated lysines on histone 4 (H4K5ac and H4K8ac) may also be recognized by the bromodomain of TRIM24 [36].Therefore, ubiquitination and degradation of target proteins of TRIM24, such as p53, on active chromatin may curtail transcription activity, while the ligase may serve as a transcription co-activator for transcription factors that are not ubiquitination targets [37,38]. The direct interaction of TRIM24 with mutant p53 may lead to its degradation, which would promote the normal activity of the wild-type allele or change the conformation of the mutated protein to a wild-type configuration able to function as a transcription regulator of the p53 transcriptome, as observed in mutant p53-transformed embryonic stem cells [39]. The finding that TRIM24 is well expressed in a TCGA colorectal cancer cohort and that overexpression is independent of the presence and type of *TP53* mutations suggests that TRIM24 is not regulated by p53 and could serve as a regulator of mutant p53, promoting its degradation or its normal configuration. These two alternative fates would be both tumor-suppressing by neutralizing the influence of the mutant protein, but a more detailed evaluation of the interactions of p53 and TRIM24 and their outcomes in colorectal cancer remains to be performed.

It should be noted that mRNA expression of different genes captures a variety of transcription factor activity and epigenetic regulations, including promoter methylation, which is a prevalent phenotype dominating a subset of colorectal cancers [40]. This phenotype, CIMP, is most frequently observed in right colon cancers and cancers with MSI and is associated with BRAF mutations [41]. As *TP53* mutations are less common in MSI colorectal cancers, cancers with the CIMP phenotype are enriched in the *TP53* wild-type cohort. However, mRNA expression of E3 ligases regulating p53 was not downregulated in this cohort, which is consistent with their genes not being targets of CpG island methylation and suppression.

The finding that VHL and its associated E2-conjugating enzyme, UBE2S, are the highest expressed E3 ubiquitin ligase and E2 enzymes in colorectal cancers among those frequently used in PROTAC constructs may have implications for the optimal design of effective PROTACs in these cancers. Targeting of mutant p53, for example, may be more effective with a VHL-based PROTAC instead of an MDM2-based PROTAC, given that E2 enzymes working with MDM2 are less well expressed in colorectal cancers. The less robust expression of MDM2-associated E2 enzymes UBE2D1, UBE2D2, and UBE2D3 may be an impediment to the activity of a PROTAC targeting the mutant form of p53 using MDM2 as the PROTAC construct ligase.

Beyond PROTACs targeting mutant p53 proteins for degradation, stabilizer drugs that convert the defective configuration of specific mutants to the wild-type configuration, thus restoring a more normal function, are in development [40,41]. A drug of this class, called rezatapopt (previously known as PC14586), targets the Y220C p53 mutant protein, which is one of the most frequent p53 mutations observed, occurring in about 1% of all human cancers. In colorectal cancer, the Y220C mutation appears less frequently, with 1 case in 594 (0.2%) in the TCGA cohort, 2 cases in a cohort of 619 patients (0.3%) from Dana Farber Cancer Institute (DFCI), and 5 cases in a cohort of 1134 metastatic colorectal cancer patients (0.4%) from Memorial Sloan Kettering Cancer Center [16,42,43]. Interestingly, in the early phase trials of rezatapopt, patients with common *KRAS* mutations derived less benefit from the drug and are currently excluded from participation in the phase 2 PYNNACLE trial, a fact that will further decrease the colorectal cancer population potentially eligible for the drug [44]. If proven effective in these ongoing clinical trials, rezatapopt may become a valuable adjunct in the minority of cancer patients with specific *TP53* mutations and, equally important, may pave the way for the discovery of other stabilizers to target frequent *TP53* mutations, including those at positions 175, 248, 273, and 282, in a personalized manner.

The current study has some limitations. It is based on a single cohort with publicly available data on mRNA expressions and may not represent the colorectal cancer patients of other practices. However, TCGA has collected patients from centers in the United States and across the Atlantic. The whole exome approach employed broadens the coverage of genes examined but decreases the depth of coverage, with the risk of missing alterations, especially in samples with low tumor cellularity. Although the cohort was large, with almost 600 patients, representing one of the most extensive genomic cohorts available in colorectal cancer, some subgroups with rare alterations may not have been well represented enough to derive reliable conclusions.

Targeting cancer alterations that were previously considered not targetable is increasingly becoming a reality, and mutations in p53 are the next frontier in cancer therapeutics, one that, given the key role of the normal protein, may be a breakthrough for the field. Nevertheless, an individualized approach taking into consideration the specific mutations and the genomic environment of the individual tumor will be necessary, as it is unlikely that activator drugs able to restore mutant p53 function independently of the specific mutant conformation would be discovered. In addition, PROTACs, working by destroying the mutant protein to allow the unimpeded function of the normal protein derived from the wild-type allele, could ubiquitinate an array of mutant p53 proteins. Still, these drugs would need to collaborate with intact ubiquitination machinery to be effective and would require expression and normal function of proteins of this machinery, as the examples of VHL and UBE2S illustrate.

This study describes in a comprehensive manner the landscape of all ubiquitinating enzymes involved in the regulation of p53. It also dissects the expression of E3 ligases according to the TP53 mutation status. This study offers a compilation of expressions of p53 regulators in both normal and colorectal cancer tissues at the mRNA and protein levels. The analysis of the expressions of E3 enzymes used in PROTACs and collaborating E2 enzymes provides a roadmap for drug development in this therapeutic niche. In addition, this represents a novel concept for individualized treatment that may be used for other cancers and other targets.

## Figures and Tables

**Table 1:** p53-regulating E3 ligases. RING: Really Interesting New Gene, HECT: Homologous to E6-AP C Terminus, K: Lysine, and Ub: ubiquitination. Question mark denotes unknown.

Ligase	Aliase(s)	Type	Chromosome	Cellular Location	Targeted K in p53	Type of Chain
MDM2 [23]	HDM2	RING	12q15	Nucleoplasm, cytosol	K370, K372, K373, K381, K382, K386	K48, mono-Ub
UBE3A	E6-AP	HECT	15q11.2	Nucleoplasm, cytosol	K370, K372, K373, K381, K382, K386	K48
HUWE1 [24]	ARF-BP1, Mule	HECT	Xp111.22	Nucleoplasm, cytosol	?	K48
RCHY1 [25]	PIRH2	RING	4q21.1	Nucleoplasm, cytosol	K101, K164, K292, K305, K357	K48
COP1 [26]	RFWD2	RING	1q25.1-q25.2	Nucleoplasm, cytosol	?	K48
TRIM24 [27]	TIF1A, RNF82	RING	7q33-q34	Nuclear bodies	Can target phosphorylated p53 at S15 and S20 (DNA damage induced)	K63
RNF34	CARP-1	RING	12q24.31	Perinuclear region	?	?, mono-Ub
RFFL	CARP-2	RING	17q12	Endosome membrane and plasma membrane	?	K48

**Table 2:** Expression of p53-regulating E3 ligases in normal colon and rectum epithelia. NA: not available. Data are from GTEx and the Human Protein Atlas (HPA).

Ligase	mRNA Expression Colon (nTPM)	mRNA Expression Rectum (nTPM)	Protein Expression Colon	Protein Expression Rectum
MDM2	21.8	23.4	High	High
UBE3A	32.3	28	High	High
HUWE1	47.8	22.4	Medium	Medium
RCHY1	11.7	12.8	Medium	Low
COP1	17.5	16.2	NA	NA
TRIM24	7.8	8	Medium	Medium
RNF34	18.8	15.6	NA	NA
RFFL	25.8	22.4	High	High

**Table 3:** Expression of p53-regulating E3 ligases in colorectal cancers. Some E3 ligases were assayed with more than one antibody. FPKM: fragment per kilobase exons per million reads; NA: not available.

Ligase	mRNA Expression (FPKM) Median (25th–75th Percentile)		Protein Expression			
	Colon	Rectum				
MDM2	65.4 (47.5–93.1)	56.8 (43.9–100.3)	CAB00086: 11/11 high	CAB016303: 12/12 High		CAB079977: 2/9 High, 4/9 medium, 2/9 low, 1/9 not detected
UBE3A	74.1 (62–89.5)	83.3 (69.4–95.9)	HPA039410: 8/11 medium, 3/11 low	HPA040380: 10/12 medium, 2/12 low		CAB009723: 11/11 High
HUWE1	53.9 (43.9–66.7)	58 (42.4–68.2)	HPA002548: 1/11 High, 9/11 medium, 1/11 low	CAB022718: 2/12 High, 2/12 Medium, 7/12 low, 1/12 not detected		
RCHY1	7.7 (5.7–9.8)	8 (5.7–10.3)	HPA030339: 3/12 medium, 2/12 low, 7/12 not detected			
COP1	25.9 (20.9–32)	28.1 (22.3–28.1)	NA			
TRIM24	14.1 (11.1–18.6)	15.1 (12.1–20)	HPA043495: 5/12 medium, 3/12 low, 4/12 not detected			
RNF34	27 (22.6–31.5)	26.2 (23.5–30.2)	NA			
RFFL	26.9 (19.6–35.5)	26.5 (19.2–31.6)	HPA017910: 1/12 high, 9/12 medium, 2/12 low	HPA019492: 2/11 High, 4/11 medium, 5/11 low	CAB008096: 9/12 High, 3/12 medium	

**Table 4:** Characteristics of colorectal cancers with and without *TP53* mutations from TCGA. Percentages in parentheses. GOF: gain of function; NA: not available.

Characteristic	Entire Cohort (n = 594)	*TP53* Wild Type (n = 220)	*TP53* Mutant (n = 314)	*p*-Value	*TP53* GOF Mutant (n = 94)	*TP53* Other Mutant (n = 220)	*p*-Value
Age (mean+/−SD)	66.1+/−13.4	66.5+/−13.2	65.3+/−12.8	0.29	67.1+/−12.3	64.5+/−13	0.31
Age							
≤65 years	260 (43.9)	93 (42.3)	147 (47.1)	0.28	40 (42.6)	107 (49.1)	0.32
>65 years	332 (56.1)	127 (57.7)	165 (52.9)		54 (57.4)	111 (50.9)	
NA	2		2			2	
Sex							
Male	312 (52.7)	112 (50.9)	165 (52.9)	0.66	51 (54.3)	114 (52.3)	0.8
Female	280 (47.3)	108 (49.1)	147 (47.1)		43 (45.7)	104 (47.7)	
NA	2		2			2	
Stage							
I	104 (17.9)	50 (23.2)	45 (14.8)	0.002	11 (12)	34 (16)	0.67
II	220 (37.9)	97 (44.9)	104 (34.2)		30 (32.6)	74 (34.9)	
III	170 (29.3)	45 (20.8)	106 (34.9)		36 (39.1)	70 (33.1)	
IV	86 (14.8)	24 (11.1)	49 (16.1)		15 (16.3)	34 (16)	
NA	14	4	10		2	8	
Primary location							
Colon	436 (74.1)	180 (82.2)	211 (68.3)	0.0004	58 (61.7)	153 (71.2)	0.11
Rectal	152 (25.9)	39 (17.8)	98 (31.7)		36 (38.3)	62 (28.8)	
NA	6	1	5			5	

**Table 5:** Subtype, tumor mutation burden (TMB), aneuploidy score (AS), and fraction genome altered (FGA) in colorectal cancers with or without *TP53* mutations, from TCGA. Percentages in parentheses. GOF: gain of function; NA: not available.

Characteristic	Entire Cohort (n = 594)	*TP53* Wild Type (n = 220)	*TP53* Mutant (n = 314)	*p*-Value	*TP53* GOF Mutant (n= 94)	*TP53* Other Mutant (n = 220)	*p*-Value
Subtype							
Colon GS	49 (10.7)	41 (25.3)	8 (2.7)	<0.00001	4 (4.3)	4 (1.9)	0.37
Colon CIN	226 (49.2)	53 (32.7)	173 (58.2)		50 (54.3)	123 (60)	
Colon MSI	60 (13.1)	43 (26.6)	17 (5.7)		3 (3.3)	14 (6.8)	
Colon POLE	6 (1.3)	4 (2.5)	2 (0.7)		0	2 (1)	
Rectal GS	9 (2)	7 (4.3)	2(0.7)		1 (1.1)	1 (0.5)	
Rectal CIN	102 (22.2)	12 (7.4)	90 (30.3)		33 (35.9)	57 (27.8)	
Rectal MSI	3 (0.6)	0	3 (1)		1 (1.1)	2 (1)	
Rectal POLE	4 (0.9)	2 (1.2)	2 (0.7)		0	2 (1)	
NA	135	58	17		2	15	
Mutation count							
<100	243 (46)	99 (46.3)	149 (47.5)	<0.00001	44 (46.8)	105 (47.7)	0.48
100–200	192 (36.4)	58 (27.1)	129 (41.1)		42 (44.7)	87 (39.6)	
>200	93 (17.6)	57 (26.6)	36 (11.4)		8 (8.5)	28 (12.7)	
NA	66	6					
AS							
<4	127 (21.7)	88 (40.4)	25 (8)	<0.00001	4 (4.2)	21 (9.7)	0.08
4–24	408 (69.6)	125 (57.3)	244 (78.5)		81 (86.2)	163 (75.1)	
>24	51 (8.7)	5 (2.3)	42 (13.5)		9 (9.6)	33 (15.2)	
NA	8	2	3			3	
FGA							
<0.1	134 (23)	91 (42.5)	28 (9.1)	<0.00001	6 (6.5)	22 (10.2)	0.52
0.1–0.35	300 (51.5)	92 (43)	186 (60.2)		56 (60.2)	130 (60.2)	
>0.35	149 (25.5)	31 (14.5)	95 (30.7)		31 (33.3)	64 (29.6)	
NA	11	6	5		1	4	

**Table 6:** Number of putative promoter binding sites of key colorectal cancer transcription factors in the two TRIM24 promoters. Promoter sequences were retrieved from the Eukaryotic Promoter Database (EPD, http://epd.expasy.org/epd/ (accessed on 8 January 2026)). Transcription factor binding motifs were retrieved from the JASPAR database (http://jaspar2022.genereg.net (accessed on 8 January 2026)). Numbers in parentheses represent the binding sites of each transcription factor relative to the transcription starting site.

	AP-1 (JUN:FOS)	TCF7L2	SMAD (SMAD2/SMAD3/SMAD4)	*TP53*	CDX2	HNF1A	TEAD4	TEAD1	E2F1
TRIM24_1	3 (−809, −703, −679)	1 (−802)	2 (−130, −25)	2 (−467, −318)	3 (−967, −862, −521)	4 (−779, −741, −612, −494)	0	0	1 (−219)
TRIM24_2	3 (−707, −601, −577)	1 (−700)	2 (−28, 77)	2 (−365, −216)	3 (−865, −760, −419)	4 (−677, −639, −510, −392)	0	1 (−970)	1 (−117)
UBE2S_1	2 (−907, −842)	4 (−734, −628, −549, −252)	5 (−932, −802, −773, −559, −510)	0	2 (−868, −28)	0	0	1 (−438)	2 (−681, −680)

## Data Availability

The original contributions presented in this study are included in the article. Further inquiries can be directed to the corresponding author.
